# Pharmacological and Nutraceutical Activation of Rejuvenation, Geroprotection and Cytoprotection: Proofs of Concept

**DOI:** 10.3390/cells11233786

**Published:** 2022-11-26

**Authors:** Gérard Lizard, Mohamed Hammami, Giuseppe Poli

**Affiliations:** 1 Team Bio-PeroxIL ‘Biochemistry of the Peroxisome, Inflammation and Lipid Metabolism’ (EA 7270), Université de Bourgogne, Inserm, 21000 Dijon, France; 2 Lab-NAFS ‘Nutrition-Functional Food & Vascular Health’, Faculty of Medicine, LR12ES05, University Monastir, Monastir 5000, Tunisia; 3 Department of Clinical and Biological Sciences, University of Turin, San Luigi Hospital, Orbassano, 10043 Turin, Italy

Aging is a process associated with life. It takes place at a variable rate in adults, and it is possible to distinguish chronological aging from physiological aging [1]. Physiological aging is the result of the involvement not only of intrinsic factors (genetic factors) but also extrinsic factors that vary from one individual to another [2,3]. In this context, the multifactorial role of the exposome, including environmental factors but also lifestyles and life events, is preponderant [4] (Figure 1). Consequently, defining a universal method to attenuate or prevent physiological aging, which can lead to pathological aging when associated with age-related diseases (cardiovascular, neurodegenerative and ocular diseases, osteoporosis, sarcopenia, type 2 diabetes and certain cancers) [5], is therefore a difficult challenge. It is nevertheless a major issue due to the increase in lifespan, and preventing aging, mainly pathological aging, is both a social and economic necessity [6].

Currently, advances in biology and medicine are opening up encouraging prospects for preventing aging and age-related diseases. Several avenues are possible. Acting on telomerase activity (or more precisely on the telomeric complex) which reduces the length of telomeres is one of the oldest possibilities mentioned [7,8]. Indeed, it is well established that a reduced size of telomeres leads to a reduction in lifespan by favoring certain age-related diseases [9]. We also know that a reduction in the size of telomeres contributes to cellular senescence, which in turn contributes to oxidative stress and inflammation, implicated in several pathologies of aging [10]. Identifying senolitic strategies (destruction of senescent cells) is therefore an important therapeutic challenge [11]. In addition, mitotherapy and pexotherapy, which consist of preserving the metabolic activity of certain cellular organelles, mitochondria and peroxisome, respectively, also represent promising perspectives [12,13]. Cell therapy, thanks to the use of inducible pluripotent cells (iPSC), makes it possible to consider the treatment of several diseases with important applications in regenerative medicine [14,15]. At the moment, rejuvenation is no longer a myth, and is gradually becoming a reality.

In the therapeutic arsenal available to act on aging by promoting the rejuvenation of certain organs and certain functions, and by preserving cells and tissues from various physical, chemical and/or biological attacks which can favor aging and age-related diseases, natural and synthetic molecules continue to attract interest. Thus, it has been clearly demonstrated in different animal models that resveratrol, which is a polyphenol widely associated with the Mediterranean diet, as well as some of its derivatives increase lifespan in mice and nematodes by stimulating biogenesis and mitochondrial activity involving adenosine 5′ monophosphate-activated protein kinase (AMPK), sirtuin 1 and peroxisome proliferator-activated receptor-gamma coactivator (PGC)-1 alpha (PGC1-α) [16,17,18]. Omega 3 fatty acids, tocopherols and certain phytosterols also have preventive activities on cardiovascular diseases [19,20,21]. Thus, many nutrients have the ability to delay aging [22]. Dimethyl fumarate and its major metabolite, monomethyl fumarate, have also been shown to prevent the toxicity of major cholesterol oxidation products (7-ketocholesterol and 7β-hydroxycholesterol) found at increased levels in the plasma and/or target organs of patients with cardiovascular, neurodegenerative and ocular diseases [23,24]. Currently, several data are available that show that natural and synthetic molecules are able to act on aging and on certain pathologies which result from it.

Currently, societal changes are leading to more and more interest in natural or synthetic molecules in order to consider healthy aging. In this context, it is becoming increasingly interesting to identify molecules that are capable of either promoting rejuvenation, preventing aging, or protecting against exogenous or endogenous cytotoxic compounds which can favor aging. The identification of such molecules is made possible through various studies on humans, animals or cells, and it is important to know the possible target genes of these molecules and the relevant signaling pathways in order to create applications that will improve human health.

In this Special Issue of *Cells* entitled “Rejuvenating, Geroprotective and Cytoprotective Activities of Natural and Synthetic Compounds: Proofs of Concept, Associated Mechanisms and Applications” (Guest Editor: Dr Gérard Lizard; Associated Guest Editors: Prof. Mohamed Hammami and Prof. Giuseppe Poli), twelve papers have been published (four reviews and eight research papers). These various works report the incidence of natural or synthetic molecules in the pathophysiology of several age-related diseases.

The cytoprotective effect of the taurine conjugate of ursodeoxycholic acid (TUDCA) has been carefully reviewed on the basis of recent mechanistic investigations carried out in a number of in vitro and in vivo models. The net anti-inflammatory effect exerted by TUDCA makes this physiological intermediate of bile acid metabolism a good candidate drug product in the treatment of a variety of chronic diseases whose progression is promoted by sustained inflammatory processes, including diabetes, neurodegenerative diseases and cancer [25]. 

In between pharmaceuticals and nutraceuticals, an interesting review critically analyzed the therapeutic potential of sterculic acid, a plant-derived inhibitor of stearoyl-CoA desaturase 1 (SCD1), the enzyme catalyzing the conversion of saturated fats into monounsaturated fatty acids (MUFAs). SCD1 inhibition may promote apoptosis, pyroptosis and cell cycle arrest, and thus sterculic acid could be considered in the treatment protocols of non alcoholic fatty liver disease, of cancer, in which the enzyme is often overexpressed and, last but not least, of the age-related macular degeneration [26]. 

A comprehensive analysis of the most promising natural compounds to be used as nutraceuticals in the treatment of inflammatory disease processes, with special regard to osteoarthritis, has also been provided. The main mechanistic aspects behind the pleiotropic effects of substances such as glucosamine, chondroitin sulfate and hyaluronic acid, omega-3 fatty acids, resveratrol, and curcumin were elucidated by means of sound in vitro and in vivo preclinical studies. The potential contribution of these and many other nutraceuticals to nutrigenomics was discussed [27]. 

A special focus on the beneficial effects of the polyphenols present in large amounts in the extra virgin olive oil was nicely afforded and their primary role as nutritional supplements in elderly was properly underlined by reviewing the increasing bulk of data proving their remarkable antioxidant and anti-inflammatory properties. Notably, their in vitro demonstrated effects in quenching the enterotoxicity of dietary cholesterol oxidation compounds (oxysterols), especially those derived from food cholesterol oxidation, were underlined and properly commented on [28]. 

Of note, new and informative mechanistic insights into the protective effects exerted by polyphenols against a number of cell changes, at least partly due to a cellular redox imbalance, consistently detectable in age-related diseases, were given by a bulk of original in vitro research studies. The proof of the great efficacy of three polyphenols, namely quercetin, resveratrol and apigenin, in counteracting the multifaceted cytotoxicity of 7-ketocholesterol was provided in the mouse neuronal N2a cell line, since this major oxysterol was shown to accumulate in patients with age-related diseases, in particular cardiovascular and neurodegenerative processes [29,30]. A deep mechanistic insight clearly connected the ROS quenching and reinforcement of the antioxidant defense system to the ability of the three nutraceuticals to positively modulate a number of redox-dependent signaling pathways and transcription factors, that in case of 7-ketocholesterol’s excessive accumulation ultimately would lead to different expressions of cell death, especially oxiapoptophagy, associated with several markers of oxidative stress, apoptosis and autophagy [31]. Another polyphenol, xanthohumol, of the flavonoid sub-family, is already considered as a potentially useful supplement in anti-cancer therapies, in particular against the progression of colon cancer, a type of malignant neoplasia not sometimes high resistance to chemotherapeutics. New mechanistic proofs that support the ability of this natural compound to induce apoptosis of colon cancer cells, at the same time activating post-DNA repair reactions, making the residual neoplastic cells sensitive to chemotherapy again, were obtained in a suitable in vitro model [32]. A promising curcumin derivative, GT863, was demonstrated to efficiently interfere with amyloid-β production using neuronal cells of human origin (SH-SY-5Y), acting on γ-secretase, not inhibiting its activity but rather avoiding its cleavage through the N-glycosylation of this protein subunit nicastrin. Indeed, the inhibition of N-glycosylation in neuronal cells as exerted by the GT863 curcumin derivative appears of particular interest, because of the better bioavailability shown by this compound in comparison to curcumin [33]. Polyphenols appear to be the key players in the anti-breast cancer effects disclosed by an extract of the plant Ephedra alata Dacne, tested in combination with the cisplatin therapy both in a cancer cell line and in a mouse model. All of the components of the plant extract were identified by liquid chromatography combined with mass spectrometry, revealing that about 50% of the total compounds were represented by quercetin and myricetin and their derivatives. The plant extract was shown to synergize with cisplatin in inhibiting the proliferation and enhancing the apoptosis of breast cancer cells [34]. 

Rather more related to the pre-clinical evaluation of a variety of pharmacological interventions are other interesting original studies reported in this Special Issue. Sterculic acid, independently from its known inhibitory effect on stearoyl-CoA desaturase, was proven to exert a significant anti-inflammatory and anti-proliferative role on layers of retinal pigment epithelial cells in culture, by exploiting quite a large number of properties, including the inhibition of fatty acids and steroid synthesis and the up-regulation of fatty acid degradation and β-oxidation, all original findings detected by an advanced technology, namely genome-wide transcriptomics, a promising therapeutic option in the treatment of age-related macular degeneration [35]. Mitotane, the standard drug used to treat adrenocortical carcinoma (ACC) with reasonably good results, impairs not only the adrenocortical steroid biosynthesis but also the cholesterol metabolism, with an increased production of some cholesterol oxidation products, named oxysterols. By means of gas chromatography combined with mass spectrometry, a net increase in the oxysterol 27-hydroxycholesterol (27OHC) in the plasma of ACC patients under treatment with the drug was observed. By treating the H295R adrenocortical cell line with micromolar amounts of 27OHC, a marked derangement of mitochondrial potential was observed, followed by strongly enhanced apoptosis. These findings point to an at least partial contribution of the 27OHC-induced apoptosis of ACC cells to the anti-cancer action of mitotane [36]. 

With regard to lung diseases, in particular idiopathic pulmonary fibrosis (IPF), myofibroblasts can become resistant to apoptosis in a FasL-dependent manner, because the membrane-bound FasL is cleaved from the cell surface by metalloproteinases, so that the soluble form of this protein is generated (sFasL). Interestingly, in IPF-lung myofibroblasts cultivated in the presence of metalloproteinase-7 (MMP-7) as well as in MMP-7 KO mice, the apoptosis of myofibroblasts induced by FasL was restored. These are original and promising results supporting the search for and possible adoption of suitable MMP-7 inhibitors in the treatment of IPF [37]. Finally, natural or synthetic drug products actively down-regulating the adhesion molecule CD44 were proposed as likely candidates to efficiently counteract the differentiation into mesencymal cells of the alveolar epithelial cells that survive acute lung damage, in this case the experimental lung damaged by bleomycin. Such an epithelial–mesenchymal transdifferentiation leads to lung fibrosis. The proof of concept was achieved in bleomycin-treated chimeric mice injected with CD44 knockout mesenchymal cells [38].

In conclusion, the different publications presented in this Special Issue of *Cells* entitled “Rejuvenating, Geroprotective and Cytoprotective Activities of Natural and Synthetic Compounds: Proofs of Concept, Associated Mechanisms and Applications”, brings new evidences on the interest to use synthetic and natural molecules to act on aging and to treat age-related diseases.

## Figures and Tables

**Figure 1:**
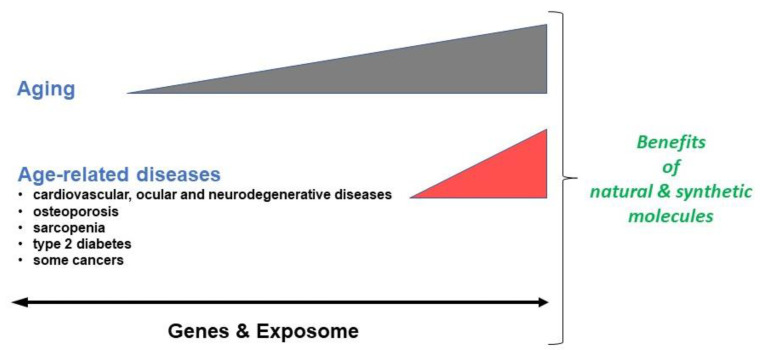
Interest of natural and synthetic molecules in the prevention and/or attenuation of aging and age-related diseases.

## References

[1] Khan S.S., Singer B.D., Vaughan D.E. (2017). Molecular and physiological manifestations and measurement of aging in humans. Aging Cell.

[2] Balaram K., Balachandran S. (2022). Psychopharmacology in the Elderly: Why Does Age Matter?. Psychiatr. Clin. N. Am..

[3] Park J., Shin D.W. (2022). Senotherapeutics and Their Molecular Mechanism for Improving Aging. Biomol. Ther..

[4] Ding E., Wang Y., Liu J., Tang S., Shi X. (2022). A review on the application of the exposome paradigm to unveil the environmental determinants of age-related diseases. Hum. Genom..

[5] Zarrouk A., Vejux A., Mackrill J., O’Callaghan Y., Hammami M., O’Brien N., Lizard G. (2014). Involvement of oxysterols in age-related diseases and ageing processes. Ageing Res. Rev..

[6] Mishra S.K., Balendra V., Esposto J., Obaid A.A., Maccioni R.B., Jha N.K., Perry G., Moustafa M., Al-Shehri M., Singh M.P. (2022). Therapeutic Antiaging Strategies. Biomedicines.

[7] Cherfils-Vicini J., Gilson É. (2020). Longevity clocks: The promoting role of telomeres?. Med. Sci..

[8] Verma A.K., Singh P., Al-Saeed F.A., Ahmed A.E., Kumar S., Kumar A., Dev K., Dohare R. (2022). Unravelling the role of telomere shortening with ageing and their potential association with diabetes, cancer, and related lifestyle factors. Tissue Cell.

[9] Boccardi M., Boccardi V. (2019). Psychological Wellbeing and Healthy Aging: Focus on Telomeres. Geriatrics.

[10] Daios S., Anogeianaki A., Kaiafa G., Kontana A., Veneti S., Gogou C., Karlafti E., Pilalas D., Kanellos I., Savopoulos C. (2022). Telomere Length as a Marker of Biological Aging: A Critical Review of Recent Literature. Curr. Med. Chem..

[11] Melo Pereira S., Ribeiro R., Logarinho E. (2019). Approaches towards Longevity: Reprogramming, Senolysis, and Improved Mitotic Competence as Anti-Aging Therapies. Int. J. Mol. Sci..

[12] Zhao Z., Yu Z., Hou Y., Zhang L., Fu A. (2020). Improvement of cognitive and motor performance with mitotherapy in aged mice. Int. J. Biol. Sci..

[13] Ghzaiel I., Zarrouk A., Essadek S., Martine L., Hammouda S., Yammine A., Ksila M., Nury T., Meddeb W., Tahri Joutey M. (2022). Protective effects of milk thistle (Sylibum marianum) seed oil and α-tocopherol against 7β-hydroxycholesterol-induced peroxisomal alterations in murine C2C12 myoblasts: Nutritional insights associated with the concept of pexotherapy. Steroids.

[14] Mora C., Serzanti M., Consiglio A., Memo M., Dell’Era P. (2017). Clinical potentials of human pluripotent stem cells. Cell Biol. Toxicol..

[15] Compagnucci C., Bertini E. (2017). The Potential of iPSCs for the Treatment of Premature Aging Disorders. Int. J. Mol. Sci..

[16] Milne J.C., Lambert P.D., Schenk S., Carney D.P., Smith J.J., Gagne D.J., Jin L., Boss O., Perni R.B., Vu C.B. (2007). Small molecule activators of SIRT1 as therapeutics for the treatment of type 2 diabetes. Nature.

[17] McCormack S., Polyak E., Ostrovsky J., Dingley S.D., Rao M., Kwon Y.J., Xiao R., Zhang Z., Nakamaru-Ogiso E., Falk M.J. (2015). Pharmacologic targeting of sirtuin and PPAR signaling improves longevity and mitochondrial physiology in respiratory chain complex I mutant Caenorhabditis elegans. Mitochondrion.

[18] Fischer N., Büchter C., Koch K., Albert S., Csuk R., Wätjen W. (2017). The resveratrol derivatives trans-3,5-dimethoxy-4-fluoro-4’-hydroxystilbene and trans-2,4’,5-trihydroxystilbene decrease oxidative stress and prolong lifespan in Caenorhabditis elegans. J. Pharm. Pharmacol..

[19] Abuajah C.I., Ogbonna A.C., Osuji C.M. (2015). Functional components and medicinal properties of food: A review. J. Food Sci. Technol..

[20] Zhang R., Han Y., McClements D.J., Xu D., Chen S. (2022). Production, Characterization, Delivery, and Cholesterol-Lowering Mechanism of Phytosterols: A Review. J. Agric. Food Chem..

[21] Ciarcià G., Bianchi S., Tomasello B., Acquaviva R., Malfa G.A., Naletova I., La Mantia A., Di Giacomo C. (2022). Vitamin E and Non-Communicable Diseases: A Review. Biomedicines.

[22] Dabrowska A., Kumar J., Rallis C. (2022). Nutrient-Response Pathways in Healthspan and Lifespan Regulation. Cells.

[23] Zarrouk A., Nury T., Karym E.M., Vejux A., Sghaier R., Gondcaille C., Andreoletti P., Trompier D., Savary S., Cherkaoui-Malki M. (2017). Attenuation of 7-ketocholesterol-induced overproduction of reactive oxygen species, apoptosis, and autophagy by dimethyl fumarate on 158N murine oligodendrocytes. J. Steroid Biochem. Mol. Biol..

[24] Sghaier R., Nury T., Leoni V., Caccia C., Pais De Barros J.P., Cherif A., Vejux A., Moreau T., Limem K., Samadi M. (2019). Dimethyl fumarate and monomethyl fumarate attenuate oxidative stress and mitochondrial alterations leading to oxiapoptophagy in 158N murine oligodendrocytes treated with 7β-hydroxycholesterol. J. Steroid Biochem Mol. Biol..

[25] Kusaczuk M. (2019). Tauroursodeoxycholate-Bile Acid with Chaperoning Activity: Molecular and Cellular Effects and Therapeutic Perspectives. Cells.

[26] Peláez R., Pariente A., Pérez-Sala Á., Larráyoz I.M. (2020). Sterculic Acid: The Mechanisms of Action beyond Stearoyl-CoA Desaturase Inhibition and Therapeutic Opportunities in Human Diseases. Cells.

[27] D’Adamo S., Cetrullo S., Panichi V., Mariani E., Flamigni F., Borzì R.M. (2020). Nutraceutical Activity in Osteoarthritis Biology: A Focus on the Nutrigenomic Role. Cells.

[28] Serreli G., Deiana M. (2020). Extra Virgin Olive Oil Polyphenols: Modulation of Cellular Pathways Related to Oxidant Species and Inflammation in Aging. Cells.

[29] Testa G., Staurenghi E., Zerbinati C., Gargiulo S., Iuliano L., Giaccone G., Fantò F., Poli G., Leonarduzzi G., Gamba P. (2016). Changes in brain oxysterols at different stages of Alzheimer’s disease: Their involvement in neuroinflammation. Redox Biol..

[30] Anderson A., Campo A., Fulton E., Corwin A., Jerome W.G., O’Connor M.S. (2020). 7-Ketocholesterol in disease and aging. Redox Biol..

[31] Yammine A., Zarrouk A., Nury T., Vejux A., Latruffe N., Vervandier-Fasseur D., Samadi M., Mackrill J.J., Greige-Gerges H., Auezova L. (2020). Prevention by Dietary Polyphenols (Resveratrol, Quercetin, Apigenin) Against 7-Ketocholesterol-Induced Oxiapoptophagy in Neuronal N2a Cells: Potential Interest for the Treatment of Neurodegenerative and Age-Related Diseases. Cells.

[32] Scagliarini A., Mathey A., Aires V., Delmas D. (2020). Xanthohumol, a Prenylated Flavonoid from Hops, Induces DNA Damages in Colorectal Cancer Cells and Sensitizes SW480 Cells to the SN38 Chemotherapeutic Agent. Cells.

[33] Urano Y., Takahachi M., Higashiura R., Fujiwara H., Funamoto S., Imai S., Futai E., Okuda M., Sugimoto H., Noguchi N. (2020). Curcumin Derivative GT863 Inhibits Amyloid-Beta Production via Inhibition of Protein N-Glycosylation. Cells.

[34] Sioud F., Amor S., Toumia I.B., Lahmar A., Aires V., Chekir-Ghedira L., Delmas D. (2020). A New Highlight of Ephedra alata Decne Properties as Potential Adjuvant in Combination with Cisplatin to Induce Cell Death of 4T1 Breast Cancer Cells In Vitro and In Vivo. Cells.

[35] Pariente A., Pérez-Sala Á., Ochoa R., Peláez R., Larráyoz I.M. (2020). Genome-Wide Transcriptomic Analysis Identifies Pathways Regulated by Sterculic Acid in Retinal Pigmented Epithelium Cells. Cells.

[36] Germano A., Rossin D., Leoni V., Iaia N., Saba L., Basile V., Puglisi S., Caccia C., Poli G., Biasi F. (2020). Involvement of 27-Hydroxycholesterol in Mitotane Action on Adrenocortical Carcinoma. Cells.

[37] Nareznoi D., Konikov-Rozenman J., Petukhov D., Breuer R., Wallach-Dayan S.B. (2020). Matrix Metalloproteinases Retain Soluble FasL-mediated Resistance to Cell Death in Fibrotic-Lung Myofibroblasts. Cells.

[38] Petukhov D., Richter-Dayan M., Fridlender Z., Breuer R., Wallach-Dayan S.B. (2019). Increased Regeneration Following Stress-Induced Lung Injury in Bleomycin-Treated Chimeric Mice with CD44 Knockout Mesenchymal Cells. Cells.

